# Adapting pharmaceutical reimbursement policies to manage spending on high-priced drugs

**DOI:** 10.1186/2052-3211-8-S1-O2

**Published:** 2015-10-05

**Authors:** Elizabeth Docteur, Ruth Lopert

**Affiliations:** 1 Elizabeth Docteur Consulting, Alexandria, VA 22314, USA; 2 George Washington University, Milken Institute of Public Health and Health Services, Department of Health Policy, 950 New Hampshire Ave, Washington, DC 20052, USA

## Problem statement

Very high-priced pharmaceuticals also known as "specialty" drugs constitute a large and growing share of total spending on health care in OECD countries. Due to the lack of therapeutic alternatives and high perceived therapeutic value of the drugs, policies used to manage pharmaceutical spending are often impractical or ineffective in the case of specialty drugs. In this environment, there is a need to reformulate existing policies or develop new ones that are best suited to managing this growing group of products in a way that is consistent with the values and objectives underlying the policy frameworks of publicly financed health systems.

## Objective

This study was undertaken to identify public policies being used internationally in the management of high-priced medicines and to evaluate their strengths and weaknesses in terms of managing costs while promoting affordable access to effective medicines.

## Methods

The authors undertook reviews of the academic research and grey literature to identify policies used in OECD countries in the management of high-cost or specialty drugs, and information on the impact of the policies used on key outcomes. The review was supplemented by information from original interviews with pharmaceutical policy experts in selected countries. The authors compiled a list of policies being used and evidence on their impact, then undertook a policy analysis aimed at classifying the policies according to common features and implementation effects.

## Results

Preliminary results from this ongoing study indicate that countries are employing a range of policy approaches in the management of high-priced pharmaceuticals, including price controls, delayed reimbursement decisions, application of utilization controls and implementation of cost sharing. However, in cases of nearly inelastic patient - as when a drug's therapeutic outcomes are perceived as very valuable with no significant market competition - specialty drug manufacturers succeed in obtaining effective sales prices at unprecedented levels. Alternative policy formulations, including those that shape the terms of the market in which buyers and sellers interact, may be necessary if policy-makers seek to change this paradigm.

## Conclusions

Optimizing goals for management of high-cost pharmaceuticals may require trade-offs across them as informed by value judgments, meaning that there is likely no single best solution. Nevertheless, information on the strengths and weaknesses of alternative policies can be useful in efforts to choose appropriate policies in the face of uncertainty.

